# 7 Impact of Chronic Alcohol Use on Fluid Resuscitation in Burn Patients

**DOI:** 10.1093/jbcr/irac012.011

**Published:** 2022-03-23

**Authors:** Sasha McEwan, Kathleen A Iles, Lori Chrisco, Joyce Pak, Chris Agala, Felicia Williams, Booker King, Rabia Nizamani

**Affiliations:** University of North Carolina at Chapel Hill, Chapel Hill, North Carolina; University of North Carolina, Chapel Hill, North Carolina; North Carolina Jaycee Burn Center, Chapel Hill, North Carolina; University of North Carolina Chapel Hill, Carrboro, North Carolina; University of North Carolina at Chapel Hill, Chapel Hill, North Carolina; UNC Jaycee Burn Center, Chapel Hill, North Carolina; UNC Jaycee Burn Center, Chapel Hill, North Carolina; UNC Jaycee Burn Center, Chapel Hill, North Carolina

## Abstract

**Introduction:**

Acute alcohol intoxication in burn patients has been associated with increased mortality, renal dysfunction and difficulty with adequate fluid resuscitation. It is less clear how chronic alcohol use, regardless of intoxication status on admission, impacts patient outcomes. In this study, we examine chronic alcohol use and both short- and long-term outcomes in burn patients.

**Methods:**

Patients were identified using an institutional burn center registry and linked to clinical data. Adults admitted from 2017 to 2020 with a total body surface area (TBSA) % above 10% and a hospital stay greater than 2 days were eligible for inclusion. A total of 298 patients were enrolled and chart review completed for admission labs and fluid administration. Alcohol use was also examined and patients were staged based on severity and chronicity of alcohol use: none/minimal, early/moderate use, and problem/severe abuse. Renal dysfunction was defined based on Acute Kidney Injury Network criteria. Linear regression was used to assess the association between alcohol use and fluid resuscitation. Multiple logistic regression was used to assess alcohol use and renal dysfunction with adjustment for confounders.

**Results:**

Compared to patients with none/minimal (NM) alcohol use and early/moderate (EM) alcohol use, patients in the problem/severe (PS) alcohol use category were older (NM 45.4 years, EM 44.1 years, PS 52.2 years; p=0.02), had larger mean TBSA burns (NM 18%, EM 14.9%, PS 23.1%; p=0.03), were more likely to have third degree burns (NM 53.8%, EM 36%, PS 72.4%; p=0.02), and more likely to have inhalation injury (NM 7.2%, EM 0%, PS 24.1%; p< 0.001). Patients in the PS category also had a significantly longer hospital length of stay (LOS) (p< 0.001), ICU LOS (p< 0.001), and ventilator days (p=0.005). Mortality was higher for the PS group (21.7%) compared to the NM (6.6%) and EM (0%) groups; p=0.001. These correlated to higher mean hospital costs for patients in the PS category compared to those in the NM category ($394,964 versus $868,126, p< 0.001). After adjusting for TBSA, patients in the PS category required more fluid resuscitation within 48 hours of admission compared to the NM category (p=0.0138), despite a lower mean admission BMI (27.1 vs 30.03, p=0.03). Although there was a trend toward increased rates of acute renal injury within 48 hours of admission in the PS group (32.7%) vs the NM group (21%), this did not reach statistical significance.

**Conclusions:**

Chronic alcohol use was associated with more severe burn injury, increased morbidity and mortality, and greater resource use. Even after adjustment for comorbidities and TBSA, chronic alcohol use resulted in a need for increased initial fluid resuscitation.

